# Macrophage Depletion by Free Bisphosphonates and Zoledronate-Loaded Red Blood Cells

**DOI:** 10.1371/journal.pone.0101260

**Published:** 2014-06-26

**Authors:** Raffaella Sabatino, Antonella Antonelli, Serafina Battistelli, Reto Schwendener, Mauro Magnani, Luigia Rossi

**Affiliations:** 1 Department of Biomolecular Sciences, University of Urbino “Carlo Bo”, Urbino (PU), Italy; 2 Institute of Molecular Cancer Research, University of Zurich, Zurich, Switzerland; 3 EryDel SpA, Urbino (PU), Italy; Medical University of Graz, Austria

## Abstract

Bisphosphonates, besides being important drugs for the treatment of various bone diseases, could also be used to induce apoptosis in macrophage-like and cancer cells. However, their activity *in vivo* is limited by a short plasma half-life and rapid uptake within bone. Therefore, several delivery systems have been proposed to modify their pharmacokinetic profile and biodistribution. Among these, red blood cells (RBCs) represent one of the most promising biological carriers. The aim of this study was to select the best performing compound among Clodronate, Pamidronate, Ibandronate and Zoledronate in killing macrophages and to investigate RBCs as innovative carrier system to selectively target bisphosphonates to macrophages. To this end, the encapsulation of the selected bisphosphonates in autologous RBCs as well as the effect on macrophages, both *in vitro* and *in vivo* were studied. This work shows that, among the tested bisphosphonates, Zoledronate has proven to be the most active molecule. Human and murine RBCs have been successfully loaded with Zoledronate by a procedure of hypotonic dialysis and isotonic resealing, obtaining a dose-dependent drug entrapment with a maximal loading of 7.96±2.03, 6.95±3.9 and 7.0±1.89 µmoles of Zoledronate/ml of packed RBCs for human, Swiss and Balb/C murine RBCs, respectively. Engineered RBCs were able to detach human and murine macrophages *in vitro*, leading to a detachment of 66±8%, 67±8% and 60.5±3.5% for human, Swiss and Balb/C RBCs, respectively. The *in vivo* efficacy of loaded RBCs was tested in Balb/C mice administering 59 µg/mouse of RBC-encapsulated Zoledronate. By a single administration, depletion of 29.0±16.38% hepatic macrophages and of 67.84±5.48% spleen macrophages was obtained, confirming the ability of encapsulated Zoledronate to deplete macrophages *in vivo*. In conclusion, RBCs loaded with Zoledronate should be considered a suitable system for targeted delivery to macrophages, both *in vitro* and *in vivo*.

## Introduction

Macrophages play a central role in the regulation of numerous biological processes: maintenance of body homeostasis by removing senescent erythrocytes, apoptotic cells, macromolecules; defense of host by ingesting and digesting microorganisms; control of other cell activities by secretion of soluble molecules involved in establishment of inflammatory responses. Unfortunately, as less desirable consequences of their action, macrophages contribute to the pathogenesis of diseases such as immunothrombocytopenia [1] and autoimmune hemolytic anemia [2] by rapidly removing and destructing autoimmune antibody-complexed blood constituents and they also play a key role in the propagation of viruses in HIV-1 and HSV-1 infections [3]–[4]. They are responsible for the destruction of non-autologous grafted cells and materials [5] and due to the release of proinflammatory cytokines, macrophages are involved in harmful inflammatory reactions such as rheumatoid arthritis [6] and sepsis [7]. Furthermore, recent studies focus on macrophage role in inflammatory processes that occur in tumor stroma and create favorable conditions for cancer progression [8]. In particular, a sub-type of macrophages recruited at tumor site(s), the tumor associated macrophages (TAMs), are suggested to have critical functions by promoting angiogenesis and metastasis, suppressing adaptive immunity and expressing growth factors and matrix proteases [9]–[10]. Therefore, transient suppression of macrophage activities might be beneficial to patients and could even be part of a therapeutic approach. A temporary decrease of this cell lineage can be achieved by treatment with bisphosphonates (BPs), as shown in *in vitro* studies on macrophage-like cells [11]–[17] and in *in vivo* studies both on tumor-bearing mice [18]–[22] and in other pathologic conditions [23]–[29]. BPs are synthetic analogues of pyrophosphate in which the P-O-P bridge has been replaced by a non-hydrolysable P-C-P bond. The presence of a nitrogen atom in the R_2_ side chain divides them into two groups with different intracellular mechanisms of action: non-amino bisphosphonates or first generation bisphosphonates and amino bisphosphonates, classified as bisphosphonates of second and third generation, in which the nitrogen atom is enclosed in a heterocyclic ring [30]. Due to their efficacy in inhibiting bone resorption by osteoclasts, BPs are currently employed for the treatment of musculo-skeletal diseases with high bone turnover such as osteopenia [31], osteoporosis [32], hypercalcemia [33], Paget’s syndrome [34], osteogenesis imperfecta [35] and bone metastases [36]. Since macrophages belong to the same cell lineage as osteoclasts and the latter represent the major targets of BPs, their efficacy in affecting macrophages can be easily understood. To be effective in macrophage depletion, bisphosphonates should be used at high doses because these molecules are characterized by a short half-life in circulation, being rapidly diverted to the bone or excreted unaltered by the kidneys with very low extra-skeletal bioavailability. However, this exacerbates toxicity problems such as nephrotoxicity, osteonecrosis of the jaws and others already occurring at clinical doses [37]–[41] and makes it necessary to protect bisphosphonates until their release and intracellular accumulation in the target cells. For these reasons, new formulations have been explored that are able to change bisphosphonate pharmacokinetic profiles, prolong plasma half-life, reduce bone accumulation and lower general toxicity. Until now, liposome-encapsulated bisphosphonates (in particular, Clodronate-loaded liposomes, such as Clodrolip [42], [43]) have been proposed for this purpose. Preclinical studies have shown the efficacy of this treatment in the depletion of macrophages associated with solid tumors (TAMs) [43], in the treatment of tumors of macrophage origin [44], in the removal of circulating monocytes for antinociceptive effects due to nerve damage [45], in the removal of Kupffer cells involved in the onset of thrombocytopenic purpura [46] and in the onset of hemolytic anemia [47], as examples of conditions in which the temporary elimination of mononuclear phagocytes could have a clinical relevance. However, such vehicles have reduced clinical application because of limitations in production technologies [48] and experienced toxicity *in vivo* [22], [49]. An alternative to liposomes is the use of erythrocytes, whose efficacy as carrier system has been extensively documented in the last years [50]–[55]. Indeed, among the systems used in drug delivery, RBCs have demonstrated a high potential as drug carriers thanks to procedures inducing the opening of nanopores on their surface which allow the entry of molecules [53]. Furthermore, the possibility to modify the membrane surface by promoting the formation of antigenic sites recognized by macrophages allows the delivery of pharmaceutical molecules to these cells [56], [57]. Alternatively, lipophilic antibody-modified RBCs could be developed to selectively reach the macrophages [58], [59]. Clodronate-loaded RBCs have already shown to be effective in macrophage depletion both *in vitro* and *in vivo* [57], [60], [61]. Given the growing interest in bisphosphonates for their clinical potential, the present study aimed at identifying the best performing bisphosphonate among Clodronate, Pamidronate, Ibandronate and Zoledronate in causing detachment of adherent human and murine macrophages and at evaluating the efficacy of the selected drug loaded into RBCs in macrophage detachment in *in vitro* studies compared to Clodrolip. Finally, engineered RBCs have been successively investigated in Balb/C mice receiving intravenous infusions of the selected bisphosphonate-loaded RBCs for the depletion of liver and spleen macrophages.

## Materials and Methods

### Human blood samples

Human blood samples in heparinized vacutainers were obtained from healthy volunteers included in the list of blood donors from the Blood Transfusion Center of the Hospital “S. Maria della Misericordia”, Urbino (PU) Italy after signing a written informed consent, under a protocol approved by the Ethical Committee of the University of Urbino “Carlo Bo” (Protocol Number: 6589, approved on June 16^th^ 2010).

### Animals

Female Swiss and Balb/C mice were purchased from Charles River Laboratories (Milan, Italy). Mice were kept under controlled conditions at 22±1°C, 12 h light/dark cycle, 60±1% humidity and 12 air changes/hour and provided with food and water *ad libitum*. Healthy mice were at least 3 months old when used in experiments. Use and care of the animals were approved by the Ethical Committee of the University of Urbino “Carlo Bo” (Permit Number: CESA 1/2013, approved on February 06^th^ 2013) and all procedures were conducted in conformity with national and European laws.

### Reagents

**Figure 1:**
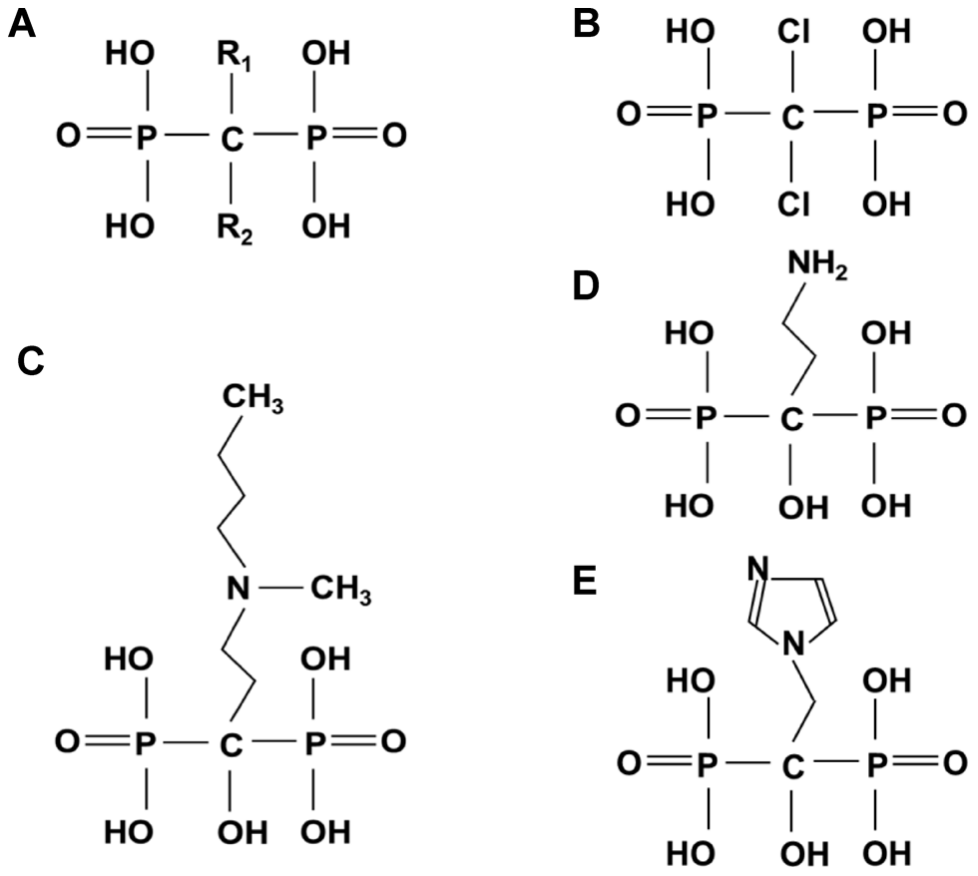
Bisphosphonates. General structure **A)**, Clodronate **B)**, Pamidronate **C)**, Ibandronate **D)**, Zoledronate **E)**.

Zoledronate used in *in vitro* experiments was kindly provided by Novartis Pharma (Basel, Switzerland). Clodronate, Pamidronate, Ibandronate were purchased from Sigma Aldrich (St. Louis, MO, USA), as well as Zoledronate used in *in vivo* studies. The structure of bisphosphonates is depicted in Figure 1. Bisphosphonate stock solutions were prepared in distilled water, adjusted to pH 7.4 and filter-sterilized prior to use. Empty liposomes and Clodrolip were prepared as described before [43]. Other reagents were purchased from Sigma Aldrich. All solutions were prepared in distilled water and filter-sterilized prior to use.

### Preparation of human and murine macrophages

Human monocyte-derived macrophages (M/M) from buffy-coats of healthy adult blood donors were obtained by density gradient separation using Lymphoprep solution (Axis-Shield PoC AS, Oslo) [57]. Monocytes were separated from lymphocytes by adherence to tissue plastic dishes or to 96-wells plastic plates (Sarstedt, Nümbrecht, Germany), overnight at 37°C in a humidified 5% CO_2_ atmosphere. Non adherent cells were removed by repeated washes with RPMI-1640 medium. Human monocyte-derived macrophages were cultured for seven days in RPMI 1640 medium, supplemented with 10 U/ml penicillin, 10 µg/ml streptomycin, 2 mM L-glutamine and 10% heat-inactivated fetal calf serum (Lonza, Milan, Italy) changed every two days. On the seventh day of culture, adherent macrophages were used for experiments. Macrophages (4×10^5^ per dish) were used for the treatment with bisphosphonate-loaded vehicles or free drug. Murine macrophages were collected from the peritoneum of Swiss mice. Cold PBS (10 ml) containing 100 IU/ml penicillin, 100 mg/ml streptomycin and 5 IU/ml heparin was injected into the abdominal cavity, the peritoneum was massaged and the peritoneal fluid with the cell suspension was collected in sterile centrifuge tubes. After centrifugation, cells were suspended in completed RPMI 1640 medium and 5×10^6^ cells were placed in culture tissue dishes and incubated for 12 h at 37°C in a 5% CO_2_ atmosphere. Peritoneal cells were gently washed with PBS several times and treated with bisphosphonate-loaded vehicles or free drugs.

### Loading of Zoledronate in human and murine RBCs

Zoledronate was encapsulated in human RBCs by a procedure of hypotonic dialysis, isotonic resealing and reannealing as previously reported [62], with some modifications. Briefly, human RBCs were washed in 10 mM Hepes (pH 7.4), containing 154 mM NaCl and 5 mM glucose, to remove leukocytes and platelets. RBCs were resuspended at 40% hematocrit (Ht) in the same washing buffer and dialyzed for 120 min against 50 volumes of 10 mM NaH_2_PO_4_, 10 mM NaHCO_3_ and 20 mM glucose (pH 7.4), containing 3 mM glutathione and 2 mM ATP (dialysis buffer) using a tube with a cut-off of 12–14 kDa. The osmolality of the buffer was about 60 mOsm, whereas the RBCs reached about 80 mOsm at the end of dialysis. All these procedures were performed at 4°C. After this step, 50 µmol of drug were added to each milliliter of dialyzed RBCs, which were then incubated for 30 min at room temperature under gentle mixing. Resealing of RBCs was obtained by adding PIGPA (5 mM adenosine, 100 mM inosine, 2 mM ATP, 100 mM MgCl_2_, 0.194 M NaCl, 1.606 M KCl and 35 mM NaH_2_PO_4_, pH 7.4) in order to reestablish isotonicity and incubating the resealed cells at 37°C for 25 min. RBCs were washed three times with washing buffer and further processed to increase their recognition by macrophages as described below. As control, unloaded (UL) RBCs, *i.e.* RBCs submitted to the loading procedure but without bisphosphonate addition, in the *in vitro* experiments were used. To encapsulate the drug in murine RBCs, blood was collected from CO_2_-anesthetized Swiss and Balb/C mice by puncture of the retro-orbital sinus in heparinized capillary tubes. Then the same procedure performed for human RBCs was carried out, except that a different dialysis buffer (15 mM NaH_2_PO_4_ and 15 mM NaHCO_3_ instead of 10 mM) and a different hematocrit (60% Ht) were used. At the end of dialysis, Swiss and Balb/C RBCs reached about 100 and 125 mOsm, respectively (an extended dialysis up to 150 min was subsequently needed for the reduction to 100 mOsm by Balb/C RBCs, thus increasing loading efficiency). To load increasing amounts of Zoledronate, dialyzed Swiss RBCs were incubated with 10–25–50 mM Zoledronate, whereas dialyzed Balb/C RBCs were incubated with 20–40–60 mM Zoledronate and processed as previously described.

### Targeting of Zoledronate-loaded RBCs to macrophages

Targeting of bisphosphonate-loaded RBCs to macrophages was obtained essentially by promoting the formation of band 3 clusters, which are recognized by the immune system as senescence antigens [63] and consequently opsonized by autologous antibodies. In detail, the loaded RBC suspension (10% Ht) was incubated with 1.0 mM ZnCl_2_ and treated with 1.0 mM bis(sulfosuccinimidyl)suberate (BS^3^) for 15 min at room temperature under gentle mixing, then washed once with buffer containing 10 mM ethanolamine (pH 7.4) and once with buffer containing 1% (w/v) bovine serum albumine (BSA). RBCs were then incubated in autologous plasma for 60 min at 37°C at a hematocrit of 20% to induce IgG binding, followed by washing once with buffer containing 2% (w/v) BSA and once with plain buffer. Unloaded RBCs underwent the same procedure. Zoledronate-loaded RBCs were then added to macrophages (100∶1 v/v ratio) and their ability to detach cells from culture dishes was evaluated and compared to control RBCs.

### Assessment of haematological parameters

Red blood cell number, mean corpuscular volume (MCV), mean corpuscular haemoglobin (MCH) and mean corpuscular haemoglobin concentration (MCHC) were determined by a haemocytometer (ABX MICROS, Horiba ABX SAS, Montpellier, France) before and after the drug loading procedure.

### Quantification of encapsulated-Zoledronate by LC-MS/MS

The quantitative analysis of Zoledronate was performed by Accelera S.r.l. (Nerviano, MI, Italy). To determine the amount of bisphosphonate encapsulated into RBCs, an aliquot of opsonized Zoledronate-loaded RBCs was resuspended at 10% Ht and processed as reported by *Veldboer K et al.* [64], with some modifications. Samples (50 µl) were transferred to an Eppendorf tube and added with 100 µl of 10 mM ammonium bicarbonate. After centrifugation at 2,060 g for 15 min, the supernatant was transferred to a test tube and added with 700 µl of 10 mM ammonium bicarbonate. A solid phase extraction (SPE) was used to separate Zoledronate from matrix components and perform the derivatization reaction. Anion-exchange cartridges (100 mg; Varian, Palo Alto, CA, USA) were conditioned with 1 ml methanol and 1 ml water. An aliquot of 700 µl of samples and calibration standards was then flowed through the sorbent. Cartridges were washed with 2 ml water and 1.5 ml methanol. After addition of 100 µl trimethylsilyl diazomethane (TMS-DAM, 2 M in hexanes) the flow was stopped and methanol (750 µl) was added. After 1 hour at room temperature, the tube was opened and the solution was collected. The cartridge was subsequently rinsed with methanol (500 µl) and the obtained mixture evaporated to dryness by nitrogen streaming at 40°C. The residue was reconstituted in 200 µl of a mixture of 10 mM ammonium acetate (pH 5.0) and methanol (95∶5, v/v). An aliquot of 15 µl was injected onto the analytical column. The LC-MS/MS system consisted of a hybrid triple quadrupole/linear ion trap 4000QTRAP (Applied Biosystems/MDS SCIEX, Foster City, CA, USA) mass spectrometer interfaced to a 1100 series HPLC pump (Agilent) and a CTC PAL autosampler (CTC Analytics, Zwingen, Switzerland). A binary gradient of mobile phase A (10 mM ammonium acetate, pH 5.0) and mobile phase B (methanol) was applied for the chromatographic separation. A Gemini C18 column (50×2.0 mm, 5.0 µm; Phenomenex, Castel Maggiore, Italy) was used. The analyte was ionized with an ionspray voltage of 5200 V, using 40 psi nebulizer and 40 psi heater gases at a temperature of 600°C. Detection was performed by monitoring the transition 329>203 m/z in MRM positive ion mode. The results were expressed as µmoles of Zoledronate/ml of packed (100% Ht) RBCs.

### Evaluation of macrophage detachment by Trypan Blue count

Clodronate, Pamidronate, Ibandronate and Zoledronate were added to human macrophage cultures at concentrations ranging from 0.25 to 1.0 mM. After 48 h incubation at 37°C, detached cells were collected and counted by Trypan Blue staining. The cells still remaining adherent were removed by using a scraper and counted. In subsequent experiments, human and murine macrophages were treated overnight (16 h) with Zoledronate-loaded RBCs at a ratio of 100 RBCs per macrophage. After incubation, dishes were gently washed with culture medium several times to remove the non-phagocytized RBCs. Exhausted medium was replaced and the incubation continued. At different times, macrophages were processed as above and the percentage of adherent cells compared to total adherent plus detached cells was calculated. UL RBCs, free drug, empty liposomes and Clodrolip were administered as controls.

### Evaluation of macrophage viability by MTS assay

The ability of viable human macrophages to reduce the MTS [3-(4,5-dimethylthiazol-2-yl)-5-(3-carboxymethoxyphenyl)-2-(4-sulfophenyl)-2H-tetrazolium] reagent was also assessed following treatment with the free bisphosphonates Clodronate, Pamidronate, Ibandronate and Zoledronate at concentrations ranging from 0.25 to 1.0 mM. In detail, after incubation of macrophages with BPs for 2 and 6 days the drugs were removed by repeated washings with PBS. MTS (0.4 mg/ml) reagent was added to each well and plates were placed at 37°C for 3 h. The absorbance was measured at 490 nm using a Microplate Reader (Bio-Rad, Segrate, Italy). Results are expressed as percentage of cell viability in respect to control cells.

### Stability of Zoledronate-loaded RBCs

In order to evaluate the stability of RBCs after the loading procedure and the ability to retain the encapsulated drugs, an RBC count was performed at different times (0–2-16–24 h) of incubation. In detail, Swiss mouse erythrocytes loaded with different amounts of Zoledronate were dispensed in 35 mm diameter dishes in complete RPMI medium at Hts ranging from 0.4 to 0.8% and incubated at 37°C. At scheduled times, the dishes were gently agitated to resuspend the cells and the number of RBCs was determined. Each condition was analysed in duplicate. Furthermore, after 16 h of incubation, the amount of drug inside the RBCs was quantified by LC-MS/MS analysis.

### In vivo experiments

Balb/C mice 8 weeks old and of 22–24 g body weight, were used. The ability of Zoledronate-loaded RBCs to deplete macrophages *in vivo* was assessed in healthy mice. Mice (n = 3) were injected intravenously (i.v.) with 200 µl of Zoledronate-loaded RBCs at 12.5% Ht and sacrificed after 7 days. Spleen and liver were removed and total numbers of macrophages were counted by immunohistochemistry.

### Tissue processing

The explanted organs were immediately processed according to the following protocol: fresh specimens were fixed in buffered 4% formaldehyde for 14–16 hours and dehydrated by increasing gradients of ethanol. Samples were then degreased twice in xylene and embedded in paraffin at 58°C. Semi-thin sections (3 µm) were produced with a rotary microtome (TOP Rotary S-130, Pabisch, Milan, Italy).

### Immunohistochemistry

The sections were deparaffinized twice in xylene and rehydrated in a descending ethanol series. After removal of the embedding material, endogenous peroxidases were blocked with 3% H_2_O_2_, 30% methanol in Tris-buffered saline (TBS, pH 7.4). Then, sections were rinsed in TBS, incubated in buffered citrate solution (pH 5.9) for 30 min at 96°C and rinsed with TBS. Unspecific binding was blocked with 20% normal goat serum (Millipore, Billerica, MA, USA). Rat anti-mouse antibody against F4/80 (AbD Serotec, Bio-Rad) was diluted 1∶100 in TBS plus 2% (w/v) BSA and used as primary antibody, to detect total macrophages. Primary antibody was incubated over night at 4°C, followed by the secondary goat anti-rat horseradish peroxidase-conjugated antibody (AbD Serotec) which was incubated for 1 h at room temperature. Diaminobenzidine was used as final chromogen. Sections were counterstained with haematoxylin for 7 min. Sections were dehydrated through a graded alcohol series, degreased and mounted in Eukitt. Tissue samples were observed at 40× magnification to identify positive cells. F4/80 positive cells were counted in 10 randomly chosen fields/slide. As negative control, the staining procedures were carried out without primary antibody, whereas rat IgGs were used as isotype control. No immunoreactivity was observed in both conditions. The sections were also haematoxylin and eosin (H&E) stained, to check the general morphology of specimens.

### Statistical analysis

Data are presented as Mean ± Standard Deviation (SD). Statistical comparisons were performed by one-way analysis of variance (ANOVA) with Bonferroni post-hoc test, for multiple comparisons, or by *unpaired t* test with Tukey post-hoc test, for comparisons between two groups. Differences were considered significant at P values <0.05.

## Results

### Identification of the most efficient bisphophonate

**Figure 2:**
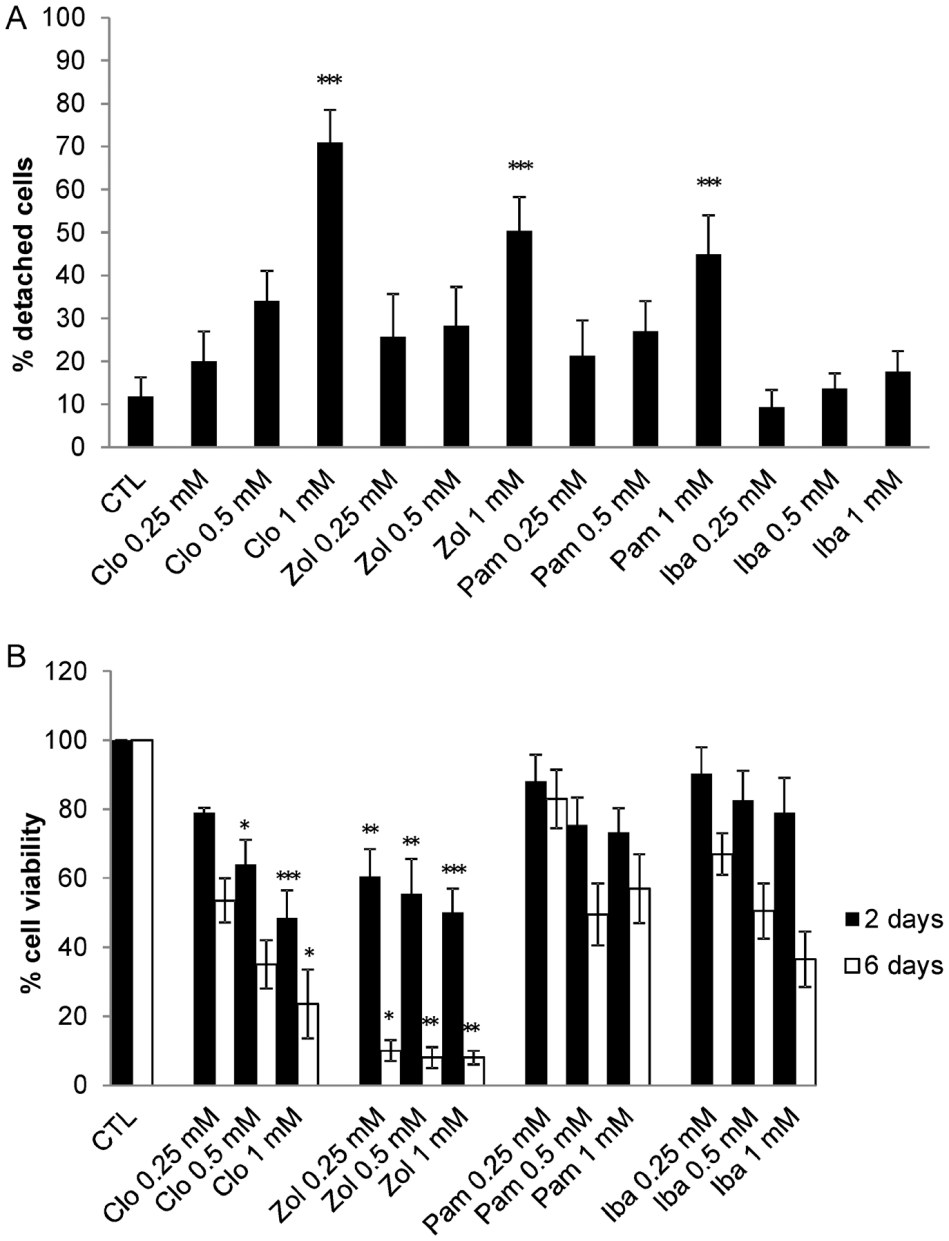
Effect of free bisphosphonates on human macrophage cultures. Count of adherent and non-adherent macrophages by Trypan Blue staining after 48 h of continuous treatment with four different bisphosphonates at concentrations ranging from 0.25 to 1.0 mM **A)**. MTS evaluation of cell viability after 48 h and 6 days of continuous treatment with four different bisphosphonates at concentrations ranging from 0.25 to 1.0 mM **B)**. Results are mean ± SD of at least 3 experiments performed in duplicate. CTL = controls; Clo = Clodronate; Zol = Zoledronate; Pam = Pamidronate; Iba = Ibandronate. *** p<0.001, **p<0.01 and *p<0.05 by ANOVA analysis with Bonferroni post-hoc test.

To identify the most efficient bisphosphonate able to detach macrophages Clodronate, Pamidronate, Ibandronate and Zoledronate were administered for 48 h to human macrophage cultures at concentrations ranging from 0.25 to 1.0 mM. A dose-dependent detachment was obtained (Fig. 2a) reaching 71±8% by Clodronate, 45±9% by Pamidronate, 18±5% by Ibandronate and 50±8% by Zoledronate at 1.0 mM BP. In MTS cytotoxicity experiments, the viability after 48 h treatment was 49±8% for Clodronate, 73±7% for Pamidronate, 79±10% for Ibandronate and 50±8% for Zoledronate (Fig. 2b). The MTS evaluation was further prolonged up to 6 days, assessing a vitality of 24±10% for Clodronate and 8±2% for Zoledronate (Fig. 2b). On the basis of the overall results, Zoledronate was chosen as reference drug in subsequent tests, aimed at evaluating the ability of RBC-encapsulated bisphosphonates to detach macrophages.

### Loading and quantification of Zoledronate in human and murine RBCs

Zoledronate loading was carried out both in human and in murine RBCs. Zoledronate was encapsulated by a procedure of hypotonic dialysis and isotonic resealing as described above. The quantification of the intra-cellular drug concentration, evaluated after the loading and opsonization phase, was made by LC-MS/MS analysis, resulting in 7.96±2.03 and 6.95±3.9 µmoles of Zoledronate/ml of packed RBCs in human and Swiss RBCs, respectively. Furthermore, varying the amount of drug added to dialyzed Swiss mouse RBCs (10, 25, and 50 mM Zoledronate) increasing encapsulation was obtained resulting in 1.87, 3.36 and 5.57 µmoles Zoledronate/ml of packed RBCs, respectively.

Since the *in vivo* efficacy of encapsulated Zoledronate was tested in Balb/C mice, the loading experiments were optimized in Balb/C RBCs. Balb/C RBCs were dialyzed against the hypotonic buffer for 120 min and successively incubated with different amounts of drug (20, 40 and 60 mM Zoledronate), obtaining an increasing encapsulation rate of 0.16±0.02, 1.88±0.07 and 2.46±1.03 µmoles Zoledronate/ml of packed RBCs, respectively, which was lower than that observed in Swiss mouse erythrocytes. This result is probably due to the higher cell osmolarity obtained at the end of the dialysis step in Balb/C RBCs (125 mOsm), compared to the Swiss cells (100 mOsm). To increase the extent of encapsulated drug, the dialysis was prolonged up to 150 min, reaching an osmolarity value comparable to Swiss RBCs (100 mOsm). Incubating these RBCs with 50 mM of Zoledronate, corresponding to the highest drug concentration that allows to remain below 150 mOsm (limit of osmolarity at which the transient membrane pores are opened) a loading of 7.0±1.89 µmoles Zoledronate/ml of packed RBCs was achieved. The results of loading of Balb/C RBCs are summarized in Table 1.

**Table 1:** Zoledronate loading in Balb/C RBCs.

Dialysis time (min)	mOsm at the end of dialysis	mM Zol during incubation	µmoles Zol/ml packed RBCs
120	125	20	0.16±0.02
120	125	40	1.88±0.07
120	125	60	2.46±1.03
150	100	50	7.00±1.89

The results are mean ± SD of three experiments.

### Evaluation of human and murine hematological parameters

For all loading experiments, the assessment of mean corpuscular indices of loaded RBCs was performed. The mean cell volume (MCV), the mean hemoglobin content and concentration (MCH and MCHC) of RBCs from untreated blood (NT) were compared to those of human and murine cells submitted to the loading procedure. The results summarized in Table 2 show that the dialysis process in hypotonic medium led to a reduction of cell volume and to a decrease in hemoglobin content. For human RBCs, the reduction was more consistent, whereas for murine RBCs the differences in respect to untreated cells were less pronounced, evidencing a minor susceptibility of murine RBCs to membrane modifications. Moreover, at the end of the loading procedure, Balb/C RBCs presented a lower MCV than Swiss mouse RBCs.

**Table 2:** Mean corpuscular indexes of RBCs.

		MCV (µm^3^)	MCH (pg Hb/RBC)	MCHC (g/dl)
**Human**	NT	87±5	31±4	36±3
	Loaded	68±7	23±10	33±11
**Swiss**	NT	46±1	19±1	41±1
	Loaded	42±8	18±4	42±1
**Balb/C**	NT	47±2	16±0.3	34±2
	Loaded	39±1	15±1	38±2

The results are mean ± SD of three experiments. NT = not-treated RBCs.

### Efficacy of human Zoledronate-loaded RBCs in macrophage detachment

**Figure 3:**
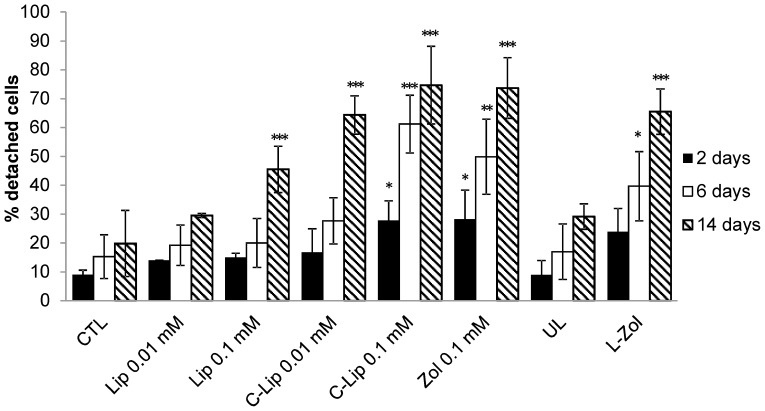
Effect of free and encapsulated bisphosphonates on human macrophage cultures. Count of adherent and non-adherent macrophages by Trypan Blue staining after 48 h, 6 days and 14 days after removal of drugs. Results are mean ± SD of at least 3 experiments performed in duplicate. CTL = controls; Lip = empty liposomes; C-lip 0.01, 0.1 mM = Clodronate-liposomes at 0.01 and 0.1 mM concentration, respectively; Zol 0.1 mM = Zoledronate at 0.1 mM concentration; UL = unloaded RBCs; L-Zol = RBCs loaded with Zoledronate to a final concentration of 7.96±2.03 µmoles/ml of packed RBCs. *** p<0.001, **p<0.01 and *p<0.05. by ANOVA analysis with Bonferroni post-hoc test.

Human RBCs loaded with Zoledronate to a final concentration of 7.96±2.03 µmoles/ml of packed RBCs were administered overnight to cultured human macrophages. At different times after RBC removal (2, 6 and 14 days), the percentages of macrophages adherent to plastic dishes were evaluated (Fig. 3). The results show that a single administration of Zoledronate-loaded RBCs is able to significantly detach macrophages in a time-dependent manner leading to 66±8% of cell detachment after 14 days treatment. When UL RBCs were administered, 29±4% of detached cells was observed and 20±11% for controls, respectively. On day 14, a significant reduction in adhesion ability following free Zoledronate (74±11%) administration was observed. The number of detached macrophages after treatment with different amounts of empty liposomes and Clodrolip was also evaluated. As expected, the extent of detachment was time- and concentration- dependent, confirming results previously reported by *van Rojien N et al.* [65]. After 14 days treatment with 0.1 mM Clodrolip 75±13% of macrophages lost adhesion. However, addition of 0.1 mM empty liposomes caused a detachment of 46±13%.

### Evaluation of murine macrophage detachment

**Figure 4:**
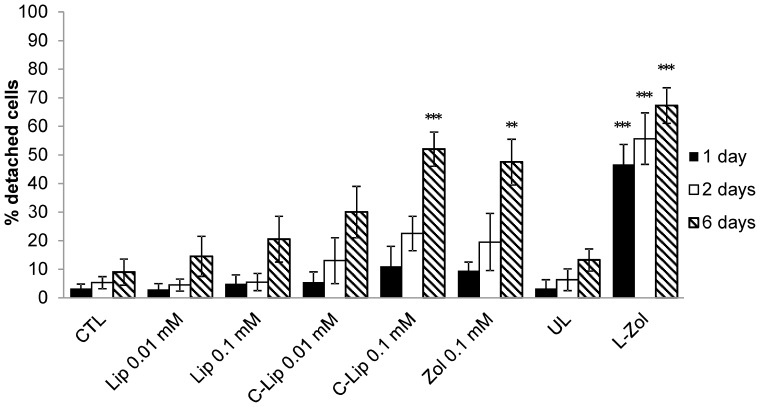
Effect of free and encapsulated bisphosphonates on Swiss mouse macrophage cultures. Count of adherent and non-adherent macrophages by Trypan Blue staining after 1, 2 and 6 days after removal of drugs. Results are mean ±SD of at least 3 experiments performed in duplicate. CTL = controls; Lip = empty liposomes; C-lip 0.01, 0.1 mM = Clodronate-liposomes at 0.01 and 0.1 mM concentration, respectively; Zol 0.1 mM = Zoledronate at 0.1 mM concentration; UL = unloaded RBCs; L-Zol = RBCs loaded with Zoledronate to a final concentration of 6.95±3.9 µmoles/ml of packed RBCs. *** p<0.001, and **p<0.01 by ANOVA analysis with Bonferroni post-hoc test.

**Figure 5:**
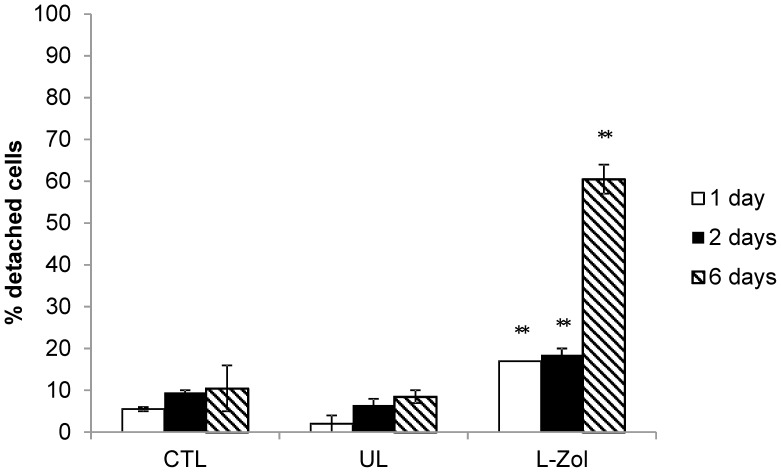
Effect of free and encapsulated bisphosphonates on Balb/C mouse macrophage cultures. Count of adherent and non-adherent macrophages by Trypan Blue staining after 1, 2 and 6 days after removal of drugs. Results are mean ±SD of 3 experiments. CTL = controls; UL = unloaded RBCs; L-Zol = RBCs loaded with Zoledronate to a final concentration of 7.0±1.89 µmoles/ml of packed RBCs. **p<0.01 by ANOVA analysis with Bonferroni post-hoc test.

**Figure 6:**
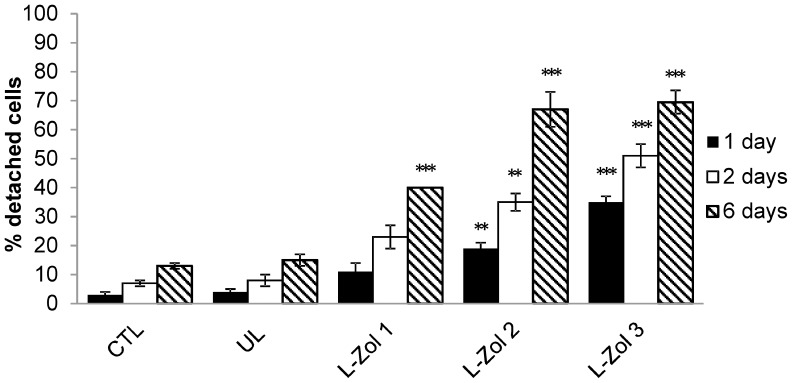
Effect of different Zoledronate concentrations in loaded erythrocytes on Swiss mouse macrophage cultures. Count of adherent and non-adherent macrophages by Trypan Blue staining, 1, 2 and 6 days after removal of drugs. Results are mean ±SD of 3 experiments. CTL = controls; UL = unloaded RBCs; L-Zol 1, 2, 3 = RBCs loaded with Zoledronate to a final concentration of 1.87, 3.36 and 5.57 µmoles/ml of packed RBCs, respectively. *** p<0.001 and **p<0.01 by ANOVA analysis with Bonferroni post-hoc test.

The ability of murine bisphosphonate-loaded RBCs to reduce the number of plastic-adherent peritoneal macrophages was evaluated. Swiss mouse RBCs containing 6.95±3.9 µmoles Zoledronate/ml of packed RBCs were added overnight to murine peritoneal macrophages and the number of adherent macrophages was evaluated after 1, 2 and 6 days. Clodrolip was also administered and as controls UL RBCs, empty liposomes and free Zoledronate were used. As shown in Figure 4, the loss of cell adhesion was concentration- and time-dependent for all tested conditions. It should be noted that macrophages treated with Zoledronate-loaded RBCs showed a significant and marked detachment already after 24 h, highlighting the effectiveness of Zoledronate-loaded RBCs. The highest macrophage detachment was observed 6 days after administration of encapsulated Zoledronate when 67±6% cell detachment was reached. When free Zoledronate was used, the adherence of macrophages to plastic dishes was affected to a lesser extent and 48±8% detachment was obtained at 0.1 mM concentration. A good activity was also seen with 0.1 mM Clodrolip with 52±6% detachment. By administering UL RBCs only 13±4% detachment was observed, a value slightly higher than that registered with untreated control macrophages (9±5%). Six days after RBC removal similar results were obtained with Zoledronate encapsulated in Balb/C RBCs at a final concentration of 7.0±1.89 µmoles/ml of packed RBCs (Fig. 5). Starting from this evidence, to better characterize the effect of engineered RBCs, macrophage detachment after administration of RBCs loaded with increasing amounts of Zoledronate (1.87, 3.36, and 5.57 µmoles/ml of packed RBCs) was evaluated in Swiss mouse macrophages. The results (Fig. 6) showed that by increasing the concentration of added drug, the ability of Zoledronate-loaded RBCs to promote detachment increased as well. This result was particularly evident 1 and 2 days after removal of RBCs, while after 6 days the difference was less pronounced.

### Stability of Zoledronate-loaded RBCs

To evaluate a possible loss of drug due to cell lysis, a stability test was carried out on Swiss mouse RBCs loaded with different amounts of Zoledronate (1.87, 3.36 and 5.57 µmoles/ml of packed RBCs) by counting, as an indirect evaluation of hemolysis, the number of intact RBCs during 0–24 h incubation. The results reported in Table 3 highlight that at increasing incubation times (0–2–16–24 h) no change in erythrocyte number following the loading procedure could be detected, suggesting that the drug was essentially retained by the loaded cells. This result was confirmed by the quantification of intracellular Zoledronate: after 16 h of incubation (time during which phagocytosis of Zoledronate-loaded RBCs was allowed), 103±15% of loaded drug was detected inside the RBCs. Moreover, the different amounts of encapsulated drug did not modify RBC stability, compared to UL RBCs, showing no effect of Zoledronate on RBC integrity.

**Table 3:** Stability of Zoledronate loaded-RBCs.

	Time	0 h	2 h	16 h	24 h
**UL**	**RBCsx10^6^/µl**	0.21	0.21	0.21	0.22
	**Ht**	0.8	0.8	0.8	0.8
**L-Zol 10 mM**	**RBCsx10^6^/µl**	0.13	0.14	0.13	0.13
	**Ht**	0.5	0.5	0.5	0.5
**L-Zol 25 mM**	**RBCsx10^6^/µl**	0.16	0.16	0.17	0.16
	**Ht**	0.6	0.6	0.6	0.6
**L-Zol 50 mM**	**RBCsx10^6^/µl**	0.1	0.1	0.11	0.1
	**Ht**	0.4	0.4	0.4	0.4

RBC count at different times of incubation after the loading procedure. RBCsx10^6^/µl = millions of RBCs/µl; Ht = hematocrit; UL = unloaded RBCs; L-Zol 10, 25, 50 mM = RBCs incubated with 10, 25 and 50 mM of Zoledronate, respectively.

### In vivo experiments

**Figure 7:**
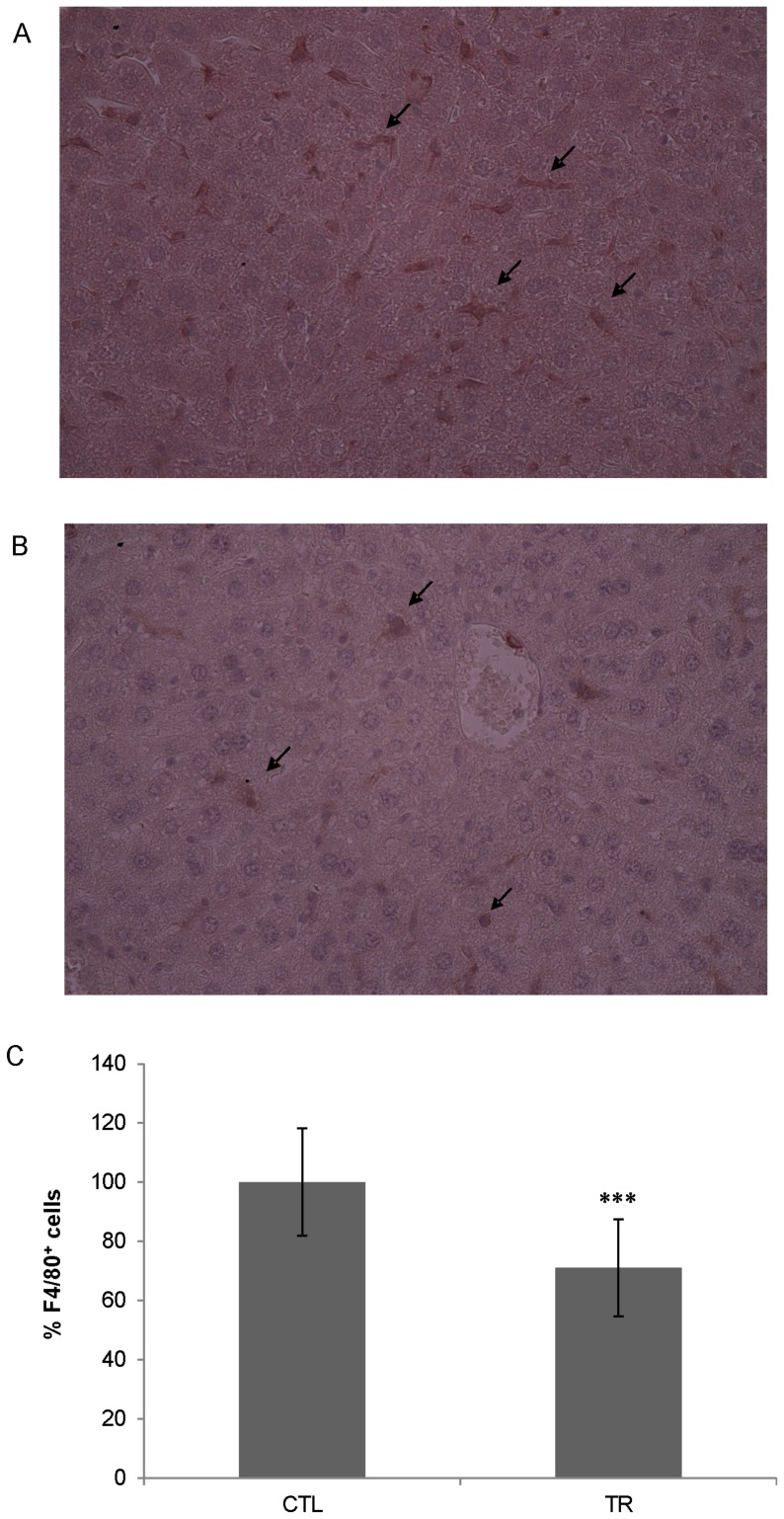
Liver immunohistochemical examination. Healthy Balb/C mice (n = 3) received intravenously 59 µg of Zoledronate by loaded RBCs. Seven days post treatment the livers were removed and processed by immunohistochemistry. Liver sections from control **A)** and treated **B)** mice were incubated with anti-F4/80 antibody. Magnification 40×. The number of macrophages was evaluated counting 10 randomly selected fields/slide and the results were expressed as percentage in relation to untreated controls **C)**. The arrows point at some F4/80 positive macrophages. CTL = controls; TR = treated mice. Control vs. treated mice, P<0.001 by *unpaired t* test with Tukey post-hoc test.

**Figure 8:**
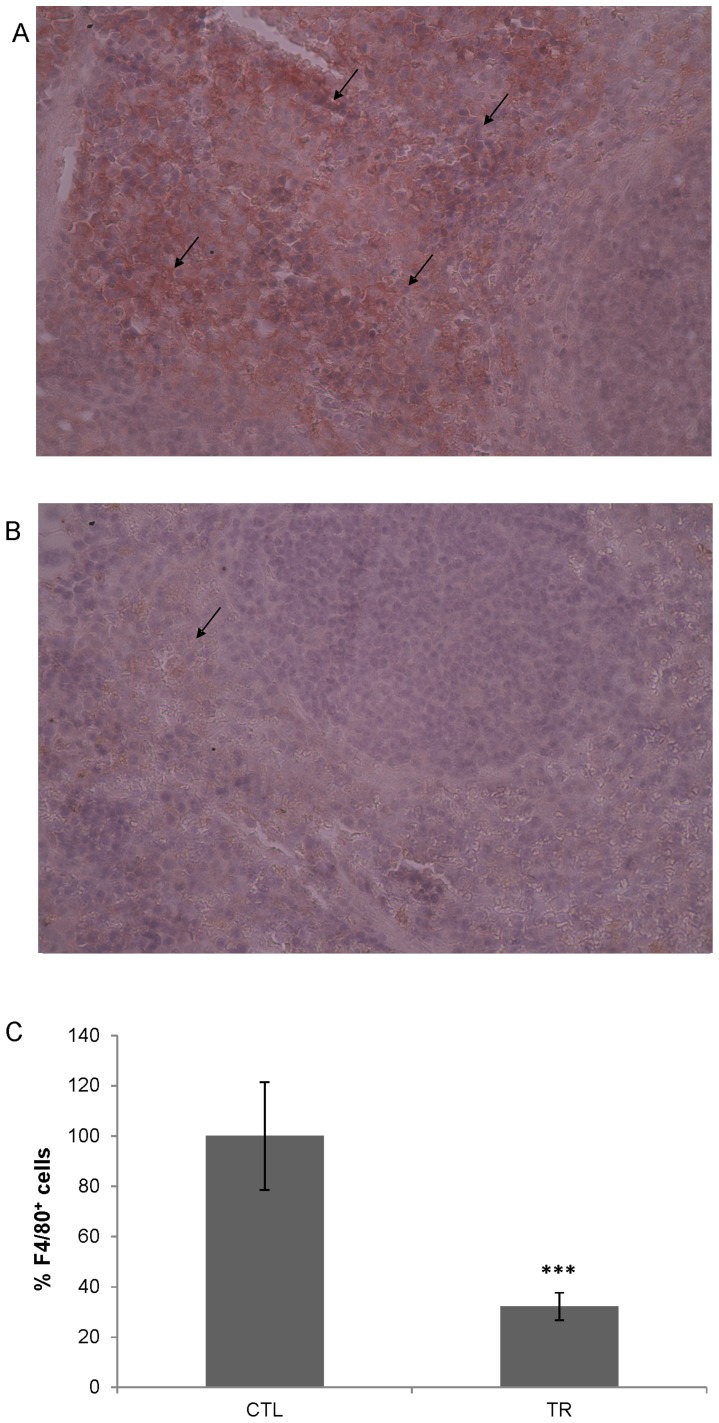
Spleen immunohistochemical examination. Healthy Balb/C mice (n = 3) received intravenously 59 µg of Zoledronate by loaded RBCs. Seven days post treatment, the spleens were removed and processed by immunohistochemistry. Spleen sections from control **A)** and treated **B)** mice were incubated with anti-F4/80 antibody. Magnification 40×. The number of macrophages was evaluated counting 10 randomly selected fields/slide and the results were expressed as percentage respect to untreated controls **C)**. The arrows point at positive zones. CTL = controls; TR = treated mice. Control vs. treated mice, P<0.001 by *unpaired t* test with Tukey post-hoc test.

To determine the *in vivo* effectiveness, Zoledronate-loaded RBCs were infused in healthy mice receiving i.v. 200 µl of RBC suspension at 12.5% Ht (corresponding to 59 µg Zoledronate). Seven days after treatment, mice were sacrificed. Spleen and liver were removed and paraffined sections were prepared as described. Sections were stained by immunohistochemical techniques to reveal total tissue macrophages and F4/80 positive cells were counted in 10 randomly chosen fields/slide. By a single Zoledronate-loaded RBC administration, a significant reduction of 29.0±16.38% (P<0.001) in hepatic macrophages (Fig. 7) and of 67.84±5.48% (P<0.001) in spleen macrophages (Fig. 8) was obtained, compared to untreated controls, thus confirming the ability of encapsulated Zoledronate to deplete macrophages.

## Discussion

Transient macrophage depletion could be a valid approach for the treatment of several clinical pathologies and literature data [11]–[29] suggest that bisphosphonates are suitable to reach this goal. Until now, liposome-encapsulated Clodronate has documented its efficacy *in vitro* and in preclinical investigations for the transient suppression of macrophage functions [42], [43]. However, liposome technology shows some limitations linked to industrial applicability [48] and toxic effects in mice [22], [49]. Thus, a safe delivery system to selectively target bisphosphonates to macrophages is still needed and an alternative approach could be represented by the use of autologous engineered RBCs. In fact, an abundant literature [see reviews 50–55] demonstrates that RBCs present unique features that make them ideal vehicles for drug delivery. They are readily available in large quantities, biocompatible when autologous RBCs are used, and completely biodegradable. They also have a prolonged life span in circulation, possess a great ability to retain encapsulated drugs and they can be loaded with a variety of molecules. Furthermore, the morphological, immunological and biochemical properties of loaded RBCs are similar to those of native cells. Thus, they can be used as carriers for sustained drug release or for targeted delivery to macrophages by exploiting their natural fate.

Our first aim was to identify the best performing bisphosphonate among Clodronate, Pamidronate, Ibandronate and Zoledronate in inducing cell detachment and death in primary human macrophage cultures, in order to select the best compound to encapsulate into RBCs. To this end, both the effect on cell adhesion and the effect on cell viability were evaluated. Clodronate, Pamidronate and Zoledronate have shown to significantly decrease the ability of macrophages to adhere to plastic dishes. Zoledronate appeared to be the most active molecule in reducing macrophage viability at all tested concentrations and thus it was the drug of choice for the RBC loading experiments. Since Zoledronate is a non-diffusible highly negatively charged molecule, it can easily be loaded into erythrocytes through a procedure which causes a transitory opening of pores in the RBC membranes, enabling Zoledronate to remain confined within the cells once pores are resealed. It is worth mentioning that the results discussed here are from Zoledronate-loaded RBCs obtained by a manual loading procedure based on hypotonic hemolysis, as usually done when small volumes (<10 ml) of blood are available. Zoledronate-loaded RBCs were modified by induction of Band 3 clusters in order to produce cells that could be phagocytized by macrophages. A similar approach has already been used to develop Clodronate-loaded RBCs, whose efficacy in depleting macrophages had been observed both in *in vitro* and *in vivo* studies [57], [60], [61]. Since our comparative data with BPs administered as free drugs showed that Zoledronate was more efficient than Clodronate, the ability of Zoledronate-loaded RBCs to detach or deplete macrophages was tested both *in vitro* and *in vivo*. In addition, it is also important to point out that Zoledronate is a latest generation bisphosphonate with numerous clinical applications. In particular, growing interest comes from its use as anti-neoplastic agent able to induce apoptosis in TAMs and to induce their repolarization to a tumor-fighting phenotype [19], thus proving to be a drug of great therapeutic interest.

Therefore, the number of human monocyte-derived macrophages remaining adherent to plastic culture dishes, at different incubation times following treatments with Zoledronate-loaded RBCs, was determined. The results showed that a single overnight (16 h) administration of RBCs was able to significantly reduce in a time-dependent manner the cell’s ability to adhere, reaching 66±8% macrophage detachment 14 days post treatment. This result was higher than that obtained with Clodronate-loaded erythrocytes (39±6%), prepared following the same procedure used to encapsulate Zoledronate, confirming previous work (data not shown) [54]. Moreover, it is noteworthy that by administering cells loaded with 7.96±2.03 µmoles of Zoledronate/ml of packed RBCs an intra-macrophage bisphosphonate concentration of approximately 0.15 mM can be expected, considering that on average 1.2 RBCs per macrophage were phagocytized [62]. When 0.1 mM of free Zoledronate was added as control, reduced adhesion ability of macrophages was also observed at a level comparable to that obtained using engineered RBCs. Nevertheless, it should be emphasized that this compound, because of its toxicity and serious side effects, cannot be administered as free drug in the clinic at the elevated concentrations utilized in this study. In fact, the dosage for Zoledronate for the treatment of bone metastases is 4 mg administered intravenously every 3–4 weeks, corresponding approximately to a peak serum concentration of 1.41 µM [66]. Clodrolip administration again confirmed its efficacy in macrophage detachment, showing results comparable to those obtained by Zoledronate-loaded RBCs.

Since the following step was to test *in vivo* the ability of such engineered RBCs to promote spleen and liver macrophage depletion, similar experiments were also performed using peritoneal murine macrophage cultures. At first, *in vitro* experiments were carried out in RBCs isolated from Swiss mice, obtaining results comparable to experiments with human RBCs loaded with drug.

Finally, with the aim to test this proposed biotechnological approach in a preclinical Balb/C murine model of pathologies which could benefit from transient macrophage depletion, the effect of Zoledronate-loaded RBCs has also been confirmed both *in vitro,* using peritoneal macrophages from Balb/C mice, and *in vivo* in the same murine strain. With regard to the *in vivo* results, a marked reduction of spleen macrophages (67.84±5.48%), compared to untreated animals, was observed by immunohistochemical analysis 7 days after administration of erythrocytes, highlighting the *in vivo* validity of this approach. It is noteworthy that this depletion was obtained following the administration of only 59 µg/mouse of Zoledronate by loaded RBCs and that it was similar to the effect previously reported in C57BL/6 mice [57] in which 220 µg of encapsulated Clodronate were administered, thus confirming the higher efficiency of our selected BP in *in vivo* macrophage depletion. Instead, a macrophage depletion of 29.0±16.38% was reached in hepatic tissue. This result was in line to results previously observed with Zoledronate-liposomes for which the highest uptake was recorded in spleen, followed by liver [49]. The phenomenon is probably due to physiology of the liver. Indeed, this organ is mainly supplied by portal vein which transports the blood refluent from the spleen. Thus, loaded erythrocytes, once i.v. injected, reach mostly the spleen where they are phagocytized by resident macrophages. Such blood, poor in loaded erythrocytes, subsequently flows to the liver through portal vein. Therefore less erythrocytes are available to deplete hepatic phagocytes.

In conclusion, this work represents one of few studies on bisphosphonate-induced detachment conducted on primary human and murine macrophage cultures and demonstrates that, among Clodronate, Pamidronate, Ibandronate and Zoledronate, the latter is the most active bisphosphonate and RBCs loaded with Zoledronate can be effective in the detachment and depletion of macrophages both *in vitro* and *in vivo*. The transient suppression of macrophage functions by employing Zoledronate-loaded RBCs could play an important role in various research applications and many biomedical phenomena with a high clinical potential.
